# ERBB3 methylation and immune infiltration in tumor microenvironment of cervical cancer

**DOI:** 10.1038/s41598-022-11415-1

**Published:** 2022-05-17

**Authors:** Xiaoyue Yang, Ying Chen, Mei Li, Weipei Zhu

**Affiliations:** 1 Department of Obstetrics and Gynecology, Affiliated Hospital of Jiangsu University, Jiefang Road 438, Zhenjiang, 212001 Jiangsu China; 2 Department of Obstetrics and Gynecology, The Second Affiliated Hospital of Soochow University, Sanxiang Road 1055, Suzhou, 215000 Jiangsu China; 3 Department of Pathology, Affiliated Hospital of Jiangsu University, Jiefang Road 438, Zhenjiang, 212001 Jiangsu China

**Keywords:** Biological techniques, Biotechnology, Cancer

## Abstract

ERBB3, a member of the ERBB family of receptor tyrosine kinases, plays an important role in cancer, despite its lack of intrinsic carcinogenic mechanism of cervical squamous cell carcinoma and endocervical adenocarcinoma (CESC). Research on bioinformatics methods through multi-omics, this work proves that ERBB3 gene mutation, methylation modification have extensive regulatory mechanisms on the CESC microenvironment. We found that ERBB3 is involved in carcinogenesis of cervical cancer and is not associated with its prognosis. The carcinogenic mechanism is mainly related to the suppression of the immune system between tumor infiltrating lymphocytes (TILs) and the methylation of the RNA level. Our study indicated ERBB3 is more likely to be a carcinogenic factor than a key prognostic factor for cervical cancer. Methylation of ERBB3 may work as a checkpoint immunotherapy target in CESC, DNA methylation modification of the 4480 base pair downstream of ERBB3 transcription initiation site was the highest.

## Introduction

A comprehensive genomic and molecular biology study of cervical cancer in the 2017 Cancer Genome Atlas project^1^ first proposed ERBB3 as a new Significant Mutation Gene (SMG) in cervical cancer and ERBB3 (HER3) as a therapeutic target. ERBB receptor tyrosine kinase family^2^ regulates a variety of biological processes, including cell proliferation, migration, invasion, and survival^3,4^. The family comprises four members: ERBB-1, also known as epidermal growth factor receptor (EGFR) HER-1, ERBB-2 (HER-2), ERBB-3 (HER-3), and ERBB-4 (HER-4). High expression or aberrant activation of epidermal growth factor receptor (EGFR) is related to tumor progression and therapy resistance^5^ across cancer types^6,7^, including CESC^8^. ERBB3 plays a key role in driving the proliferation and survival of cancer-causing cells in cervical tumors^9,10^. ERBB3 is the least studied member of the ERBB family. However, recent evidence supports the key role of ERBB3 in cell transformation and tumor malignancy^11,12^.

Persistent high-risk HPV infection has been recognized as a carcinogen of cervical cancer^13^. Recent research shows that hyper-methylation of host genes is common in precancerous and cancerous lesions and can be used as an independent risk biomarker as HPV infection. DNA methylation is an early event of cervical cancer^14,15^. Abnormal DNA methylation is an early event in tumorigenesis. The study of disease-specific methylation markers can provide new ideas for cancer screening, diagnosis and treatment^16^. In vitro laboratory tests showed that in the evolution of cervical cancer, the degree of methylation was related to the degree of cervical lesions^17^. The overall methylation level increased with the carcinogenesis process, and the increase of methylation level would increase the severity of cervical diseases^18,19^.

Recent studies have focused on the role of RNA m6A modification in tumorigenesis and development^20,21^. RNA modification has become a hot spot in the field of epigenetic transcriptomics after the rise of DNA and histone modification. N6 methyladenosine (m6A) plays an important role in the internal epigenetic modification of eukaryotic mRNA in human cancer. This post transcriptional RNA modification is dynamic and reversible and is regulated by methylase, demethylase and protein that preferentially recognize m6A modification.The m6A regulators were highly expressed in tumor tissue^22^, recent deeper studies have demonstrated epigenetic regulation of immune responses^23,24^. Nevertheless, the underlying effect of RNA N6-methyladenosine (m6A) modifications on tumor microenvironment cell infiltration remains elusive. In Esophageal Squamous Cell Carcinoma^25^, the m6A regulators are related to the tumor immune microenvironment (TIME), and their copy-number alterations will dynamically affect the number of tumor-infiltrating immune cells. At the same time, the m6A methylation regulator may be a key mediator of PD-L1 expression and immune cell infiltration and may strongly affect the TIME of ESCC. The expression pattern of m6A regulatory factor is significantly correlated with the prognosis and antitumor immune response of acute myeloid leukemia, and may be a potential target and biomarker of immunotherapy^26^. The research about the mechanism proves that low m6A score was characterized by increased mutational burden, immune activation, and survival rates.

M6A RNA modification plays a role in chemoradiotherapy resistance of cervical cancer: FTO enhances chemoradiotherapy resistance by targeting B-chain protein^27^. FTO also interacts with the transcription of E2F1 and myc to promote the proliferation and migration of cervical cancer cells^28^. At present, m6A RNA modification in cervical cancer is mainly aimed at treatment resistance and cancer cell metastasis, but there are few studies on carcinogenicity and tumor immune microenvironment.

At present, there is no unified understanding about which gene methylation detection can effectively predict or early detect precancerous lesions. The research on molecular markers of cervical cancer methylation is still in the early stage. The existing research shows us the prospect of clinical application of methylation. New methylation markers will become a useful tool for accurate diagnosis of cervical cancer.

Based on 22 m6A regulators, this study comprehensively evaluated the methylation modification patterns of 607 cervical cancer samples, systematically studied the correlation between methylation modification patterns and immune cell infiltration in tumor microenvironment, and sought more effective and accurate immunotherapy strategies.

## Materials and methods

### Data

The TCGA data involved in this study are downloaded from University of California Santa Cruz (UCSC) Cancer Browser (http://xena.ucsc.edu) with 10 GTEx normal samples; 3 TCGA adjacent samples; TCGA tumor samples. Methylation analysis data mainly include: (1) IIIumina Infinium Human Methylation450K BeadChip methylation data. We take the basic beta value for analysis; (2) IIIumina HiSeq 2000 RNA sequencing data. We download the level3data in TCGA, which is standardized by log2 (FRKM + 1).

### Gene expression analysis

Gepia2 (http://gepia2.cancer-pku.cn)^29^ was used to compare the expression of ERBB Family and m6A regulators^30^. UALCAN (http://ualcan.path.uab.edu) was used to study the correlation between target gene and clinical data in CESC^31^.

### Reverse transcription and quantitative real-time polymerase chain reaction (qRT-PCR)

The Commercial product MecDNA-HUtrC007Ce01 cDNA synthesis kit (SHANGHAI OUTDO BIOTECH CO., LTD, China) was used to perform the reverse-transcription of the extracted RNA. Arrangement of cDNA microarray: 1, CASKI; 2, MS751; 3, ME180; 4, C33A; 5, AV3; 6, SiHa; 7, HeLa. OligodT primer for reverse transcription: OligodT (15) (CD106) (TIANGEN BIOTECH CO., LTD., China); dNTP Mix: Takara dNTP mixture (4019); Reverse transcription kit (Thermo Fisher): SuperScript IV Reverse Transcriptase (18090010); Fluorescence quantitative PCR kit (Takara): SYBR^®^ Premix Ex Taq™ II (Tli RNaseH Plus) (RR820Q); Sealing film: Axygen PlateMax Ultraclear Sealing Film (UC-500); Instrument: Roche 480II; plate centrifuge: XiangYi L-530. The fold change of gene expression was calculated by 2 − (ΔCtexperimental group − ΔCtcontrol group). β-actin was used as an internal control and primers are as follows: ERBB3-Forward: 5ʹ-GACCCAGGTCTACGATGGGAA-3ʹ; ERBB3-Reverse: 5ʹ-GTGAGCTAGGTCAAGCGAG-3ʹ; Human β-actin-Forward: 5ʹ-GAAGAGCTACGAGCTGCCTGA-3ʹ; Human β-actin-Reverse: 5ʹ-CAGACAGCACTGTGTTGGCG-3ʹ (Product length: 191 bp).

### RNA m6A methylation analysis data

The literature related to m6A RNA methylation was searched in the National Center for Biotechnology Information (NCBI) literature database (http://www.ncbi.nlm.nih.gov/ncbisearch/). After extensive reading of the literature, 22 m6A regulators that have been clearly identified in our study, including m6A methyltransferase (writer): METTL14, METTL3, METTL16, WTAP and VIRMA; m6A demethylase (eraser): ALKBH5, FTO; and m6A binding proteins (reader): YTHDF3, HNRNPA2B1, HNRNPC, YTHDF2, YTHDF1, YTHDC1, YTHDC2, IGF2BP2, IGF2BP1, RBM15B, CBLL1, ZC3H13, ZCCHC4. CBioPortal (http://www.cbioportal.org/)^32,33^ is used to analyse Genomic multiomics data of m6A regulators in CESC.

### Genetic alteration analysis

After logging into the cBioPortal web, in the ‘Quick Selection’ section, we selected ‘TCGA Pan Cancer Atlas Studies’ and entered ‘ERBB3’ to query the gene change characteristics of ERBB3. The frequency, mutation type and Copy Number Change (CNA) results of CESC in TCGA were observed in the ‘Cancer Types Summary’ module. The information of ERBB3 mutation sites can be displayed in the protein structure diagram and three-dimensional structure diagram through the ‘Mutations’ module. Kaplan–Meier diagram with logarithmic rank p value was also generated by the ‘Comparison’ module (Fig. 2).

### Immune infiltration analysis

Tumor development and treatment are closely related to the immune system in the tumor microenvironment. In order to promote the comprehensive study of tumor immune interaction, through TISIDB (http://cis.hku.hk/TISIDB) to analysis the association between methylation of ERBB3 and immune characteristics of TCGA cancer types, such as lymphocytes, immunomodulators and chemokines, was calculated in advance. In TISIDB, we cross-validated the role of interest genes in tumor immune interaction through literature mining and high-throughput data analysis^34^.

### The Human Protein Atlas Gene set enrichment analysis database

The “Human Proteome” chapters provide a knowledge-based analysis and entry into the Human Protein Atlas (https://www.proteinatlas.org/) from different defined transactions of the human tissue proteome. Analysis of four genes RNA-seq expressed in 291 cervical cancer samples, The RNA-seq data is reported as average FPKM (number Fragments Per Kilobase of exon per Million reads).

### GO and KEGG analysis

Using the Database for Annotation, Visualization, and Integrated Discovery (DAVID)^35^ (https://david.ncifcrf.gov/), Gene ontology (GO) and Kyoto Encyclopedia of Genes and Genomes (KEGG) pathway enrichment analysis based on co-expressed genes (https://www.kegg.jp/), p < 0.05.

### Survival prognosis analysis

We used the “survival map” module of gepia2 to obtain the Overall Survival (OS) and Disease-Free Survival (DFS) significance map data of four genes in ERBB family of CESC in all TCGA. To assess correlations between gene expression level and OS/DFS, patients were classified into low and high mRNA expression subgroups using median expression (50%) as the cut-off. The hypothesis was tested by log rank test, and the survival map was obtained through the “survival analysis” module of gepia2. The correlation between overall survival time and m6A regulators expression is measured using Kaplan–Meier Plotter Database (https://kmplot.com/analysis/)^36^.

### Correlation between molecular and methylation degree of DNA methylation sites

Data from TCGA RNA-seq data in Level 3 HTSeq-FPKM format in CESC (cervical squamous cell carcinoma and adenocarcinoma) project and methylation data from illumina human methylation 450. RNAseq data in FPKM (fragments per kilobase per million) format is converted into TPM (transcripts per million reads) format and log2 conversion is performed.

### Statistical data

Statistical analysis on the TCGA data was performed with R software (version 3.6.0). Differentially expressed genes (DEGs) were identified using R package of mainly ggplot2 version 3.3.3. The ClusterProfiler package was used for enrichment analysis and visualization. In the previous analysis, except specifically mentioned, p value less than 0.05 was considered statistically significant.

## Results

### Gene differential expression and clinical significance of ERBB3 in CESC

**Figure 1:**
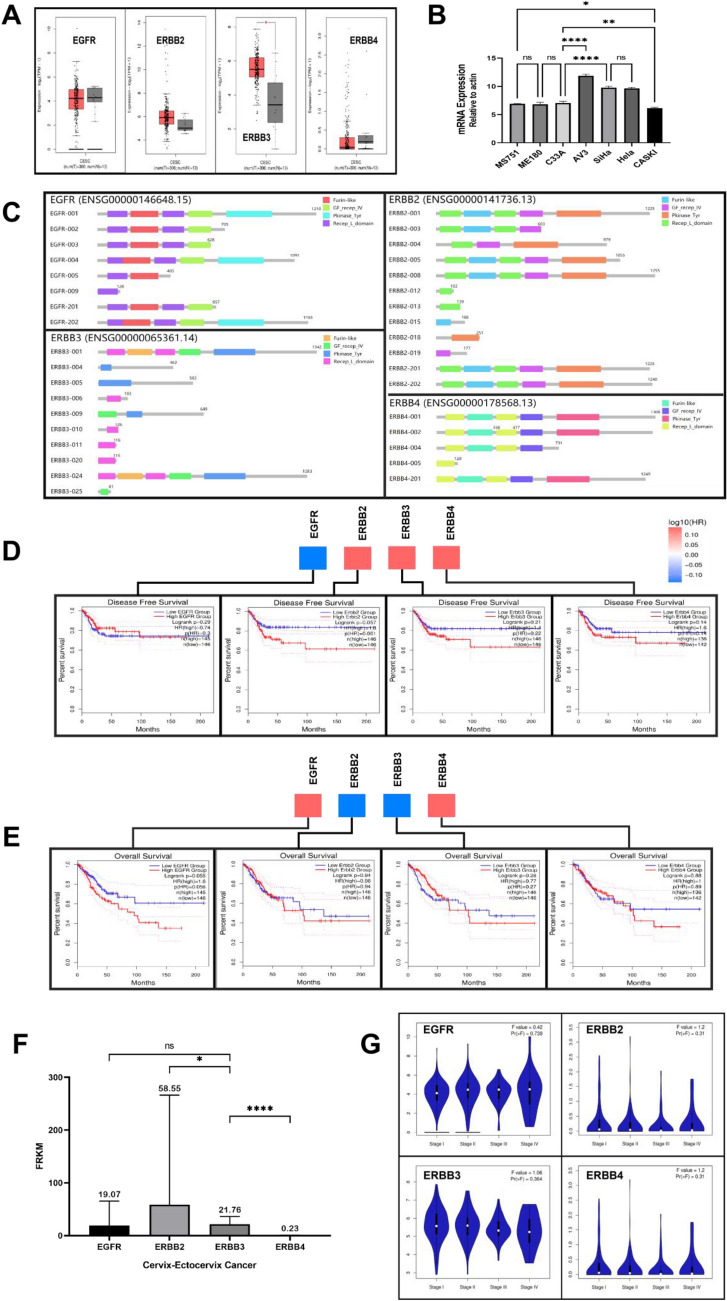
(**A**) The expression of EGFR, ERBB2, ERBB3, ERBB4 in different tumors or specific tumor subtypes in TCGA and GTEx databases analyzed by TIMER2. (**B**) ERBB3 expression in seven different cell lines was examined by real-time PCR. All PCR data were calculated relative to β-actin and represent the average ± SD of triplicate samples. (**C**) Isoform structure of EGFR, ErbB2, ErbB3, ErbB4. PFAM domains are indicated in color. (**D)** The disease free survival analyses based on the cancer type and cancer subtype showed the significant prognostic impact of four genes in CESC: EGFR (p = 0.29), ErbB2 (p = 0.057), ErbB3 (p = 0.21), ErbB4 (p = 0.14). (**E**) The overal survival analyses based on the cancer type and cancer subtype showed the significant prognostic impact of four genes in CESC: EGFR (p = 0.055), ErbB2 (p = 0.94), ErbB3 (p = 0.28), ErbB4 (p = 0.88). (**F**) EGFR, ERBB2-4 RNA-Seq data generated by the TCGA project from 291 cervical cancer samples is reported as average FRKM. (**G**) Analyze expression of four genes in CESC by clinical stages.

We first compared the expression pattern of EGFR, ERBB2, ERBB3, ERBB4 in CESC tumor and normal tissues. ERBB3 obtain a significant difference in CESC (Fig. 1A). The mRNA levels of different primary cervical cancer cell lines indicate that ERBB3 is highly expressed in cervical malignant cell lines dominated by adenocarcinoma: AV3, Hela (Fig. 1B). Further research on pathological types and HPV typing, we found that in the three types of cells with higher expression of ERBB3, there was no statistical difference between SiHa and HeLa, while the statistical difference between SiHa and C33A significantly (p < 0.0001). The difference between the two cell lines is mainly HPV infection. The low expression of ERBB3 in HPV(–)-C33A inspired us to study the significance of ERBB3 and HPV in the carcinogenicity of cervical cancer in the future.

At the same time, GEPIA2 provided the structural map of the isomeric protein domain^37^ based on Pfam prediction to show the structural differences among the isomers (Fig. 1C). Figure 1D–F analyze the clinical significance of the ERBB family. The DFS Prognostic (Fig. 1D) and OS rate (Fig. 1E) of EGFR, ERBB2, ERBB3 and ERBB4 have no significance (p > 0.05). Figure 1G shows the accordion diagram of each gene in cervical cancer staging. We found that the difference in the expression of the four genes between different stages of cervical cancer were not significant.

Figure 1F and Table 1 list the RNA-seq data of ERBB Family in 291 cervical cancer samples. RNA-seq data of EGFR, ERBB2, ERBB3, and ERBB4 in cervical and ectocervix tissue type reported as average FPKM (number Fragments Per Kilobase of exon per Million reads), generated by the The Cancer Genome Atlas (TCGA).

**Table 1:** The RNA-seq data of ERBB Family in 291 cervical cancer samples.

TCGA sample Id	EGFR	ERBB2	ERBB3	ERBB4
TCGA-ZX-AA5X-01A	10.8	26.6	20.2	0
TCGA-ZJ-AAXU-01A	9.7	10.3	9.7	0.1
TCGA-ZJ-AAXA-01A	8.4	25.7	26.8	0
TCGA-ZJ-AAX4-01A	21.1	31.5	22.5	0
TCGA-ZJ-A8QR-01A	18.1	20.2	25.4	0
TCGA-ZJ-A8QQ-01A	24.5	16.3	26	0
TCGA-XS-A8TJ-01A	11.6	33.7	30.4	0
TCGA-WL-A834-01A	13	29.3	17.5	0
TCGA-VS-AA62-01A	18.3	23	16.1	0
TCGA-VS-A9V5-01A	3.7	845.8	68.4	0.1
TCGA-VS-A9V4-01A	4.5	63.1	48.9	0.1
TCGA-VS-A9V3-01A	7.1	46	36.4	0
TCGA-VS-A9V2-01A	15.8	22.5	15.2	0
TCGA-VS-A9V1-01A	1	48	41.2	0
TCGA-VS-A9V0-01A	9.2	417.2	66	1.2
TCGA-VS-A9UZ-01A	2.2	27.9	29.2	1.3
TCGA-VS-A9UY-01A	581.3	16.9	13	0
TCGA-VS-A9UV-01A	5.2	18.9	13.4	0
TCGA-VS-A9UU-01A	14.8	23.3	14.4	0
TCGA-VS-A9UT-01A	3.7	24.8	4.1	2.1
TCGA-VS-A9UR-01A	9.8	51.9	9.1	0.6
TCGA-VS-A9UQ-01A	6	54.1	32.1	3
TCGA-VS-A9UP-01A	7.9	31.1	67.6	0.4
TCGA-VS-A9UO-01A	3.4	41.6	58.9	0.1
TCGA-VS-A9UM-01A	22.9	25.1	22.5	0.3
TCGA-VS-A9UL-01A	9.1	1146.3	8.9	0.7
TCGA-VS-A9UJ-01A	7.2	53.3	25.8	1.7
TCGA-VS-A9UI-01A	8.2	18.9	20.6	0
TCGA-VS-A9UH-01A	10.4	6.1	4.6	0
TCGA-VS-A9UD-01A	6.6	16.3	9.9	0
TCGA-VS-A9UC-01A	4.2	56.7	46.3	0.1
TCGA-VS-A9UB-01A	71.2	15	8.3	0
TCGA-VS-A9U7-01A	10.5	24.9	19.5	0.1
TCGA-VS-A9U6-01A	35.3	30.3	29.2	0
TCGA-VS-A9U5-01A	11.8	321.2	39.8	0
TCGA-VS-A959-01A	4.8	84.3	44.3	0.2
TCGA-VS-A958-01A	8.4	19	51.3	0
TCGA-VS-A957-01A	8.7	35.4	35.9	0.5
TCGA-VS-A954-01A	12.5	17.8	11.9	0
TCGA-VS-A953-01A	24.4	8.7	3.8	0
TCGA-VS-A952-01A	5.4	40.7	90	0.2
TCGA-VS-A950-01A	21.1	17.8	13.1	0
TCGA-VS-A94Z-01A	14.8	10.9	11.9	0
TCGA-VS-A94Y-01A	37.2	28.4	26.6	0
TCGA-VS-A94X-01A	32.5	29.3	16.4	0
TCGA-VS-A94W-01A	32.4	29.3	8.9	0
TCGA-VS-A8QM-01A	16.7	21.2	14.8	0
TCGA-VS-A8QH-01A	5.3	36.9	79.7	0
TCGA-VS-A8QF-01A	11.5	24.5	23.8	0
TCGA-VS-A8QC-01A	30.7	17.6	15.1	0
TCGA-VS-A8QA-01A	6.5	30.6	25.1	0.2
TCGA-VS-A8Q9-01A	13.2	17.7	13.1	0
TCGA-VS-A8Q8-01A	9.2	11.1	16	0
TCGA-VS-A8EL-01A	5	17.1	15	0
TCGA-VS-A8EK-01A	27.9	15.9	27.6	0
TCGA-VS-A8EJ-01A	0.9	42.2	44.6	0.6
TCGA-VS-A8EI-01A	22.3	18.1	14.8	0
TCGA-VS-A8EH-01A	10.6	18.4	17.8	0
TCGA-VS-A8EG-01A	15.2	34.5	23.9	0
TCGA-VS-A8EC-01A	5	32.2	22.7	0
TCGA-VS-A8EB-01A	32.1	17.5	13.8	0
TCGA-UC-A7PI-01A	5.3	74.2	62.9	1.2
TCGA-UC-A7PG-01A	17.6	20.3	18.5	0
TCGA-UC-A7PF-01A	17.5	13.9	13.9	0
TCGA-UC-A7PD-01A	20	13.2	10	0
TCGA-RA-A741-01A	15.3	30.5	21.9	0
TCGA-R2-A69V-01A	12.4	10.4	9.5	0.1
TCGA-Q1-A73S-01A	4.3	28.9	20.5	0
TCGA-Q1-A73R-01A	7.9	2321.8	18	2.1
TCGA-Q1-A73Q-01A	12.8	12.5	12.2	0
TCGA-Q1-A73P-01A	5.5	37.2	63.6	1.7
TCGA-Q1-A73O-01A	20.2	14.6	12.6	0
TCGA-Q1-A6DW-01A	21.7	11.2	13.8	0.4
TCGA-Q1-A6DV-01A	3.6	29.7	22.8	0.9
TCGA-Q1-A6DT-01A	62	9.8	6.4	0
TCGA-Q1-A5R3-01A	2	42.6	56.8	0
TCGA-Q1-A5R2-01A	8.1	24.1	14.4	0
TCGA-Q1-A5R1-01A	4.1	41.1	43.4	0.7
TCGA-PN-A8MA-01A	1.9	69.4	23.4	0.3
TCGA-MY-A913-01A	20.7	15	14.2	0
TCGA-MY-A5BF-01A	8.7	15.4	8	0
TCGA-MY-A5BE-01A	5	11.4	4.8	0
TCGA-MY-A5BD-01A	6.6	31.9	25.6	0.3
TCGA-MU-A8JM-01A	8.5	15.8	24.4	0
TCGA-MU-A5YI-01A	13.2	21.7	20.5	0
TCGA-MU-A51Y-01A	13.2	51.2	21.5	0.1
TCGA-MA-AA43-01A	14.9	491.7	3.8	0.2
TCGA-MA-AA42-01A	8.4	15.2	5.7	0
TCGA-MA-AA41-01A	421.6	14.4	13.4	0
TCGA-MA-AA3Z-01A	7.1	26.9	26.8	0
TCGA-MA-AA3Y-01A	18.6	8.9	4.5	0
TCGA-MA-AA3X-01A	15.4	18.7	16.4	0
TCGA-MA-AA3W-01A	18.8	16.1	13.1	0
TCGA-LP-A7HU-01A	1.4	40.6	24.1	0.6
TCGA-LP-A5U3-01A	9.4	1351.3	19.9	0
TCGA-LP-A5U2-01A	3.6	10.7	20.3	0
TCGA-LP-A4AX-01A	9.6	10.2	9.9	0
TCGA-LP-A4AW-01A	7.2	16.7	8.7	0
TCGA-LP-A4AU-01A	7.7	32.9	16.2	0.1
TCGA-JX-A5QV-01A	12	35.2	32.8	0
TCGA-JX-A3Q8-01A	1.3	24.3	28.3	0.7
TCGA-JX-A3Q0-01A	9.9	16.4	14.9	0
TCGA-JX-A3PZ-01A	31.3	14.2	7.8	0
TCGA-JW-AAVH-01A	10.5	28.1	37.5	0
TCGA-JW-A852-01A	3.1	14.9	10.2	0
TCGA-JW-A69B-01A	6.5	54.4	40.9	0
TCGA-JW-A5VL-01A	14.7	19.6	25	0
TCGA-JW-A5VK-01A	14.2	7.3	5.3	0
TCGA-JW-A5VJ-01A	38.2	20.2	12.9	0.1
TCGA-JW-A5VI-01A	15.4	35.5	23.3	0
TCGA-JW-A5VH-01A	0.3	10.8	11.5	0.6
TCGA-JW-A5VG-01A	38.8	14.5	5.3	0
TCGA-IR-A3LL-01A	8.2	5.7	13.7	0
TCGA-IR-A3LK-01A	16.7	25.1	28.7	0
TCGA-IR-A3LI-01A	1.4	27.5	37.4	0.2
TCGA-IR-A3LH-01A	12.8	13.8	5.1	0
TCGA-IR-A3LF-01A	4.1	33.4	44.9	1.1
TCGA-IR-A3LC-01A	18.2	35.7	25	0
TCGA-IR-A3LB-01A	5.6	22.4	28.3	0.7
TCGA-IR-A3LA-01A	5.4	42.4	43.3	0.1
TCGA-IR-A3L7-01A	17.6	42.1	42.7	0.4
TCGA-HM-A6W2-01A	3.1	4.2	7.7	0.6
TCGA-HM-A4S6-01A	27	25.3	16.9	0
TCGA-HM-A3JK-01A	14.8	30.3	11.3	0
TCGA-HM-A3JJ-01A	28.6	26	15.4	0
TCGA-HG-A2PA-01A	11.2	17.6	14.8	0.8
TCGA-GH-A9DA-01A	20.4	20	12.8	0
TCGA-FU-A770-01A	4.5	35.2	22.8	1.2
TCGA-FU-A5XV-01A	12.5	22.6	28.9	0
TCGA-FU-A57G-01A	6.8	33.7	17.3	3.5
TCGA-FU-A40J-01A	4.4	42.4	52.6	2.7
TCGA-FU-A3YQ-01A	22.5	17.3	22.9	0
TCGA-FU-A3WB-01A	14.4	31.1	17.8	0
TCGA-FU-A3TX-01A	11	19.7	14.4	0.1
TCGA-FU-A3TQ-01A	12.9	12.3	30.4	0
TCGA-FU-A3NI-01A	27	26.1	40	0
TCGA-FU-A3HZ-01A	0.1	3.3	11.4	0.2
TCGA-FU-A3HY-01A	25.1	20.1	20.9	0
TCGA-FU-A3EO-01A	1.4	37.3	59.8	0.4
TCGA-FU-A2QG-01A	19.1	21.2	33.9	0
TCGA-FU-A23L-01A	13.1	30.3	30.6	0.3
TCGA-FU-A23K-01A	0.2	25	16	0.1
TCGA-EX-A8YF-01A	4.4	46.9	38.2	1
TCGA-EX-A69M-01A	9.5	54.1	46.9	0
TCGA-EX-A69L-01A	12.2	30.2	17.2	0
TCGA-EX-A449-01A	1.1	21.4	19.6	1.9
TCGA-EX-A3L1-01A	34.3	20.6	12	0
TCGA-EX-A1H6-01B	4.4	20.7	31.6	0.7
TCGA-EX-A1H5-01A	9	28.8	23.9	0.2
TCGA-EK-A3GN-01A	11.8	18.2	32.2	0
TCGA-EK-A3GK-01A	5.9	66.7	42.6	0.3
TCGA-EK-A3GJ-01A	9.2	14.9	13.4	0
TCGA-EK-A2RO-01A	7.9	14.1	6	0
TCGA-EK-A2RN-01A	5.1	8.4	10.4	0
TCGA-EK-A2RM-01A	1	692.5	15.2	0
TCGA-EK-A2RL-01A	3.7	31.6	51	0.4
TCGA-EK-A2RK-01A	19.8	3.9	5.6	0
TCGA-EK-A2RJ-01A	9.6	24.5	17.1	0.1
TCGA-EK-A2RE-01A	31.1	13.7	9.3	0
TCGA-EK-A2RC-01A	22.8	27.2	13.4	0.1
TCGA-EK-A2RB-01A	19.6	18.5	17.8	0
TCGA-EK-A2RA-01A	14.5	22.4	24.9	0
TCGA-EK-A2R9-01A	11.7	25.6	14.9	0.1
TCGA-EK-A2R8-01A	12.7	16.7	35.5	0.1
TCGA-EK-A2R7-01A	2.7	20.8	23.6	0.2
TCGA-EK-A2PM-01A	14.7	5.7	2.4	0
TCGA-EK-A2PL-01A	17.9	29.4	35.9	0
TCGA-EK-A2PK-01A	3.4	26	52.8	0.1
TCGA-EK-A2PI-01A	18.1	14.5	9.1	0
TCGA-EK-A2PG-01A	20.7	12.7	12.3	0
TCGA-EK-A2IR-01A	22.3	17.4	9.8	0.1
TCGA-EK-A2IP-01A	13.4	16.1	18.4	0
TCGA-EK-A2H1-01A	17.7	6.1	3.9	0
TCGA-EK-A2H0-01A	25.2	13.8	24.6	0
TCGA-EK-A2GZ-01A	18.8	9.9	7	0.3
TCGA-EA-A97N-01A	15.1	17.2	29	0
TCGA-EA-A78R-01A	7.8	14.1	9.6	0
TCGA-EA-A6QX-01A	6.4	30.3	25.5	0
TCGA-EA-A5ZF-01A	2.4	56.3	33.2	0.1
TCGA-EA-A5ZE-01A	16.7	28.4	20.3	0.1
TCGA-EA-A5ZD-01A	17.5	35.2	26.9	0
TCGA-EA-A5O9-01A	5	20.4	68.5	0
TCGA-EA-A5FO-01A	24	19.9	13.3	0
TCGA-EA-A556-01A	1.4	18.1	14.4	3.1
TCGA-EA-A50E-01A	23.5	10.3	9.1	0
TCGA-EA-A4BA-01A	1.8	25.3	18.2	3.9
TCGA-EA-A44S-01A	19.4	22.3	21.4	0
TCGA-EA-A43B-01A	7.6	17	11.6	0
TCGA-EA-A439-01A	2.5	14.4	18.4	0.2
TCGA-EA-A411-01A	7.1	30.1	23.6	0.1
TCGA-EA-A410-01A	0.4	12	20.8	0.6
TCGA-EA-A3Y4-01A	4.7	17.6	15.6	0
TCGA-EA-A3QE-01A	19.2	20.5	28.7	0
TCGA-EA-A3QD-01A	7.2	15.7	14.6	0
TCGA-EA-A3HU-01A	22.8	11.2	13.4	0.1
TCGA-EA-A3HT-01A	15.8	19.3	14.5	0
TCGA-EA-A3HS-01A	40.1	18.9	24.8	0
TCGA-EA-A3HR-01A	20.2	40.9	19.2	0
TCGA-EA-A3HQ-01A	20.8	21.6	28.3	0.1
TCGA-EA-A1QT-01A	13.2	20.6	22.5	0
TCGA-EA-A1QS-01A	19.9	13.9	4.7	0
TCGA-DS-A7WI-01A	134.6	22.2	18	0
TCGA-DS-A7WH-01A	2.7	50.3	32.3	0
TCGA-DS-A7WF-01A	4.5	1570.1	15.7	1.2
TCGA-DS-A5RQ-01A	11.4	22.3	18	0
TCGA-DS-A3LQ-01A	7	25.3	18.4	0
TCGA-DS-A1OD-01A	9.3	25	16.1	0
TCGA-DS-A1OC-01A	32.8	30.9	16.4	0
TCGA-DS-A1OB-01A	28.7	12	14.5	0
TCGA-DS-A1OA-01A	12.3	20.6	20.3	0
TCGA-DS-A1O9-01A	17.6	7.7	4.8	0
TCGA-DS-A0VN-01A	12	9.3	10	0
TCGA-DS-A0VM-01A	6.5	18.4	8.9	0
TCGA-DS-A0VL-01A	12.3	23.9	31.9	0
TCGA-DS-A0VK-01A	55.6	24.8	21.6	0
TCGA-DR-A0ZM-01A	6	15.7	25.4	0
TCGA-DR-A0ZL-01A	312.3	29.1	18.2	0
TCGA-DG-A2KM-01A	7.2	10.1	16.1	0
TCGA-DG-A2KL-01A	16.5	16.4	16.7	0.1
TCGA-DG-A2KK-01A	6.4	42.1	18.2	0.1
TCGA-DG-A2KJ-01A	0.5	22.8	24.2	1.4
TCGA-DG-A2KH-01A	3.5	27.1	32.8	0.5
TCGA-C5-A907-01A	18.6	19.2	14	0
TCGA-C5-A905-01A	5.9	56.6	30	0
TCGA-C5-A902-01A	101.4	34.6	18.6	0
TCGA-C5-A901-01A	16	18.3	13.6	0
TCGA-C5-A8ZZ-01A	11.6	14.8	18.6	0
TCGA-C5-A8YT-01A	14.2	22.2	9.6	0.2
TCGA-C5-A8YR-01A	8	10.2	8.7	0
TCGA-C5-A8YQ-01A	27.3	61.2	22.8	0
TCGA-C5-A8XK-01A	23.4	20.9	15.3	0
TCGA-C5-A8XJ-01A	26.3	30.4	10.2	2.1
TCGA-C5-A8XI-01A	16.9	15.7	12.8	0
TCGA-C5-A8XH-01A	20.1	16.5	17.2	0
TCGA-C5-A7XC-01A	17	24.2	20.3	0.5
TCGA-C5-A7X8-01A	6.2	215.2	24.4	0.1
TCGA-C5-A7X5-01A	11.6	10.2	9.3	0
TCGA-C5-A7X3-01A	11.5	21.9	17.3	0
TCGA-C5-A7UI-01A	9.3	8.2	2.9	0
TCGA-C5-A7UH-01A	23	23.3	16.5	0
TCGA-C5-A7UE-01A	8.3	15	11.6	0
TCGA-C5-A7UC-01A	30.4	12.5	6.3	0
TCGA-C5-A7CO-01A	21.3	22.3	15.1	0.2
TCGA-C5-A7CM-01A	0.2	27.4	16.7	1
TCGA-C5-A7CL-01A	19.4	13.1	11.7	0
TCGA-C5-A7CK-01A	38.7	8.8	9.9	0
TCGA-C5-A7CJ-01A	30.7	19.7	19.1	0.3
TCGA-C5-A7CH-01A	23.1	17.8	13.2	0
TCGA-C5-A7CG-01A	10.6	23.3	25	0
TCGA-C5-A3HL-01A	24.9	22	14.9	0
TCGA-C5-A3HF-01A	6.2	27.6	48.4	0.3
TCGA-C5-A3HE-01A	2.5	36.2	38.7	0.3
TCGA-C5-A3HD-01B	43.7	15.3	16.7	0
TCGA-C5-A2M2-01A	5.7	46.9	69.2	2
TCGA-C5-A2M1-01A	2.2	23.8	34.4	1.1
TCGA-C5-A2LZ-01A	19.4	22.5	27.9	0
TCGA-C5-A2LY-01A	12.9	16.9	14	0
TCGA-C5-A2LX-01A	12.9	16.2	13.8	0
TCGA-C5-A2LV-01A	42.8	6.9	4.3	0
TCGA-C5-A2LT-01A	32	6.8	5.7	0
TCGA-C5-A2LS-01A	2.1	42.5	45	1.8
TCGA-C5-A1MQ-01A	14.7	31.4	24.8	0
TCGA-C5-A1MP-01A	15.2	37	13.1	0.1
TCGA-C5-A1MN-01A	35.1	11.8	10.4	0
TCGA-C5-A1ML-01A	18.1	14.7	10.7	0.2
TCGA-C5-A1MK-01A	14.4	23.8	15.7	0.5
TCGA-C5-A1MJ-01A	4.5	20	35.7	0.5
TCGA-C5-A1MI-01A	4.4	61.8	30.1	0
TCGA-C5-A1MH-01A	9.6	12.4	16.8	0
TCGA-C5-A1MF-01A	2.8	25.6	28.5	0.1
TCGA-C5-A1ME-01A	1.7	36.4	41.8	0.4
TCGA-C5-A1M9-01A	12.9	233.9	17.5	0
TCGA-C5-A1M8-01A	17.6	27.8	27.5	0.1
TCGA-C5-A1M7-01A	13.8	13.5	17.1	0
TCGA-C5-A1M6-01A	3.8	35.5	24.2	0.3
TCGA-C5-A1M5-01A	12.8	23.4	23.3	0
TCGA-C5-A1BQ-01C	15.3	14	13.9	0
TCGA-C5-A1BN-01B	37.1	9.7	5.1	0
TCGA-C5-A1BM-01A	27.8	12.8	10	0.5
TCGA-C5-A1BL-01A	22	12.4	12.4	0.1
TCGA-C5-A1BK-01B	5.7	21.5	14	0
TCGA-C5-A1BJ-01A	19.4	16	9.8	0
TCGA-C5-A1BI-01B	20.5	22.4	15.5	0
TCGA-C5-A1BF-01B	25.1	23	10	0.1
TCGA-C5-A1BE-01B	14.7	22.5	18.7	0
TCGA-C5-A0TN-01A	39.8	10.7	2.2	0
TCGA-BI-A20A-01A	12.4	32.8	18.2	0
TCGA-BI-A0VS-01A	6.4	10	16.2	0
TCGA-BI-A0VR-01A	14.1	19.9	10	0
TCGA-4J-AA1J-01A	16.1	699.1	17.4	0
TCGA-2W-A8YY-01A	3.8	36.2	30.9	0

The RNA-seq data is reported as average FPKM (number Fragments Per Kilobase of exon per Million reads), generated by the The Cancer Genome Atlas (TCGA).

**Figure 2:**
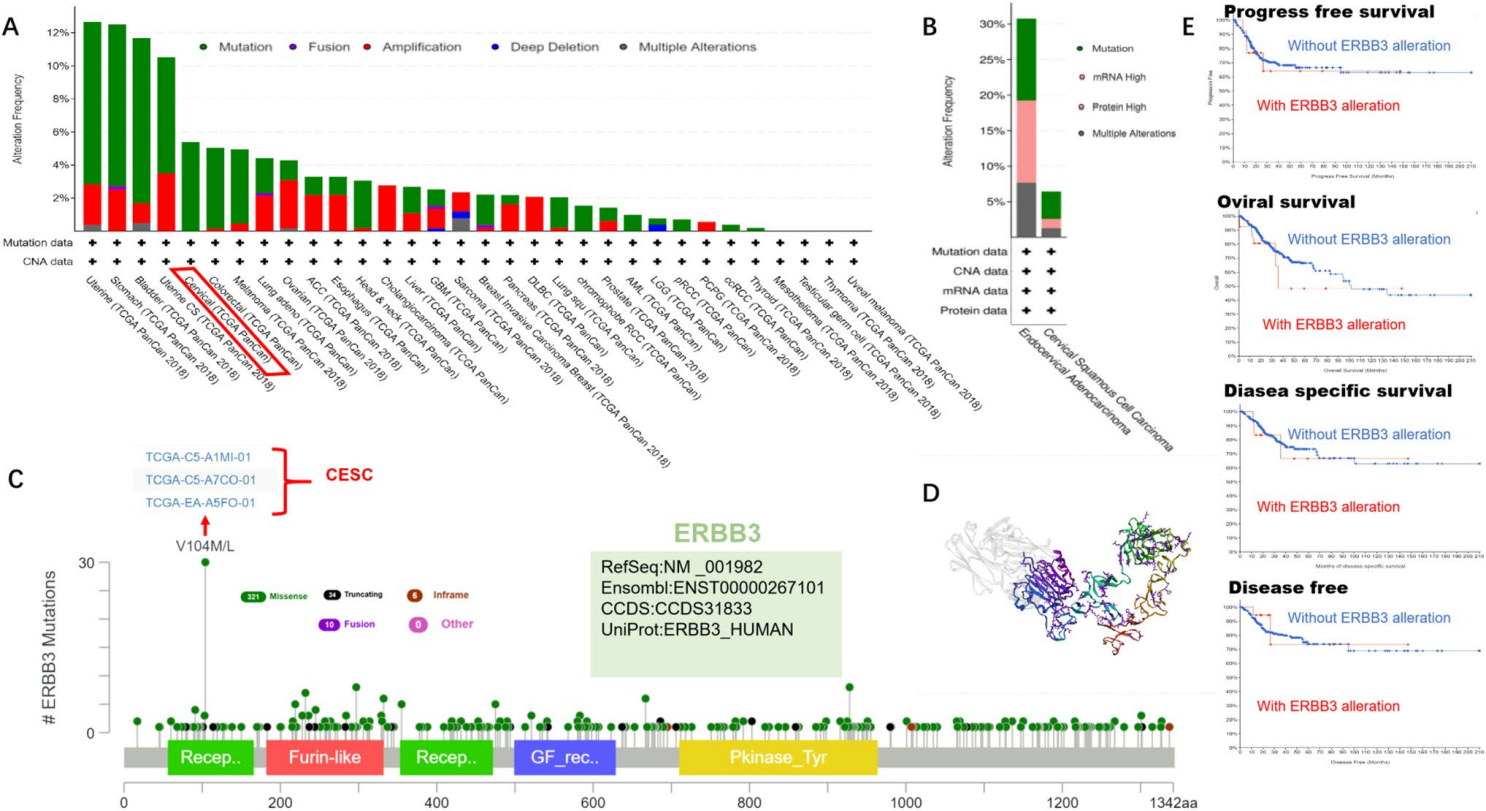
Mutation characteristics of ERBB3 in different tumors. The cBioPortal tool was used to analyze the mutation characteristics of ERBB3 in TCGA tumors. Shows the frequency of mutation type (**A**), alteration frequency of different types of cervical cancer (**B**) and mutation site (**C**). The graphical view shows the Pfam protein domains (**D**) and the positions of specific mutations. The length of the line connecting the mutation annotation to the protein is indicative of the number of samples that have the mutation. The most recurrent mutations are labeled in the graphical view. The cBioPortal tool was used to analyze the potential correlation between mutation status and progression-free, overall, disease-specific and disease-free survival of CESC (**E**).

According to the TCGA database in Fig. 2B, the alteration frequency of ERBB3 in adenocarcinoma is higher than squamous cell carcinoma. This is consistent with ERBB3 mRNA expression in different cell lines (Fig. 1B).

We showed the mutation site of V104ML with the case ID list as TCGA-C5-A1MI-01, TCGA-C5-A7CO-01, and TCGA-EA-A5FO-01 in receptor domain, which has the highest change frequency in the three-dimensional structure of ERBB3 (Fig. 2C,D). The potential correlation between mutation status and progression-free, overall, disease-specific and disease-free survival of CESC is not significant (Fig. 2E).

### m6A regulators is differently expressed in CESC cancer

We analyzed the expression of 22 major m6A RNA methylation modulators in 607 CESC patients from the TCGA dataset. This study showed that mutations in the m6A regulator of the human CESC genome were associated with pathogenesis. There were nine m6A regulatory factors significantly increased in CESCs: HNRNPC, YTHDF1, HNRNPA2B1, IGF2BP1, VIRMA, IGF2BP2, YTHDF2, RBM15, and IGF2BP3. There were seven m6A regulators with significantly reduced expression: ZCCHC4, METTL3, ZC3H13, YTHDC1, YTHDC2, METTL16, and FTO.

**Figure 3:**
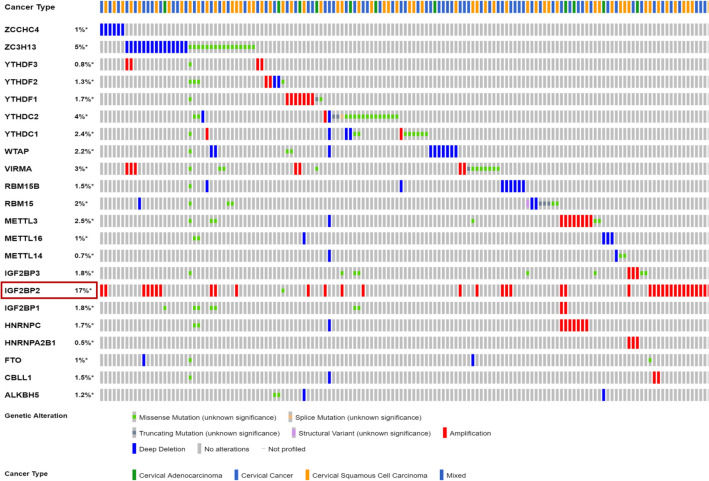
The types (Amplification, Deletion, Genetic variations and mutation) and percentages of m6A regulators alteration in 607 CESC cases using the cBioPortal database.

This study focuses on the role of m6A in CESC. Detection of genetic variation of m6A regulators in 607 patients using cBioPortal database (Fig. 3), among which IGF2BP2 displayed the highest incidence rate (17%).

**Figure 4:**
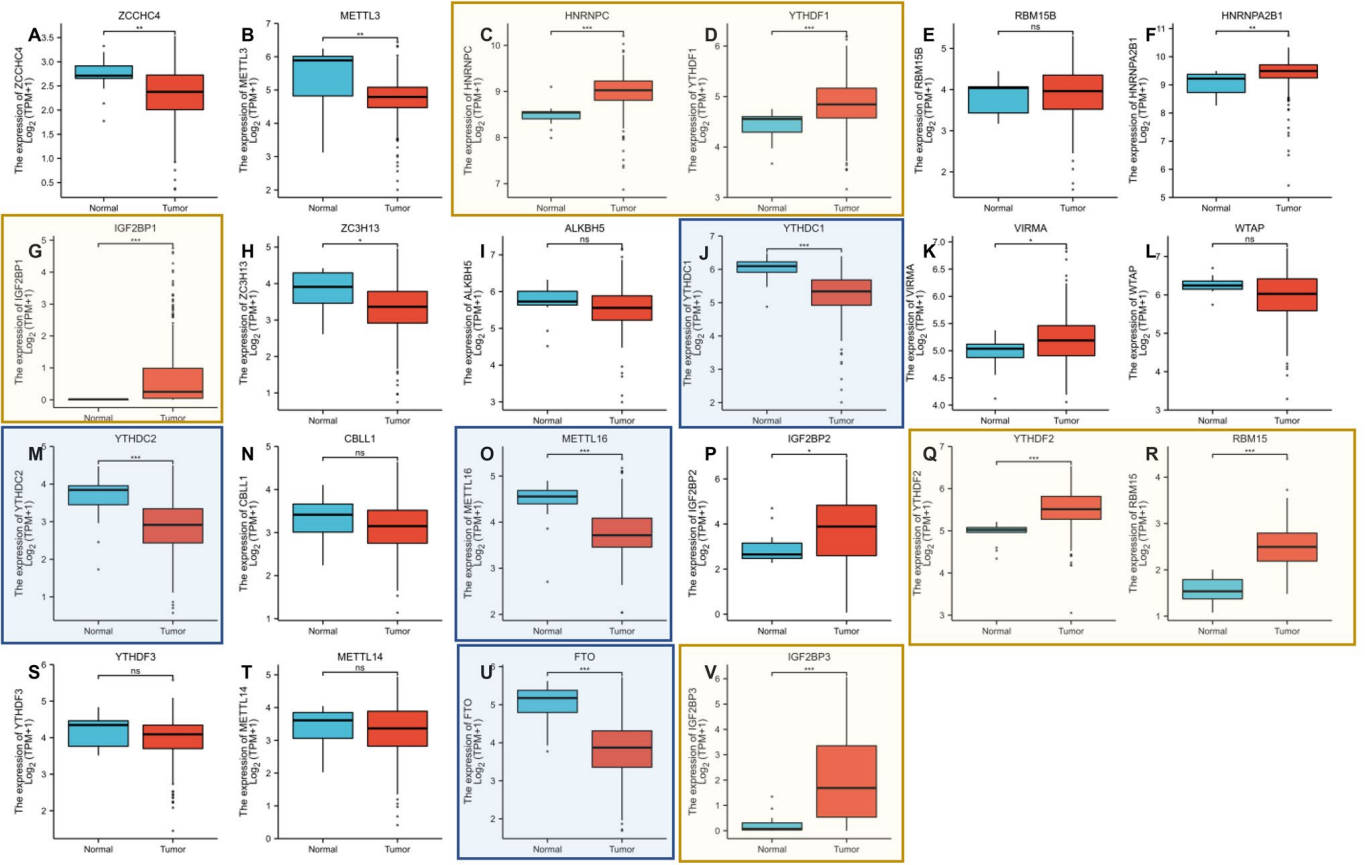
Expression profile of m6A regulators in CESC. (**A–V**) The expression levels of m6A regulators showed expression in CESC samples, including (**A**) ZCCHC4, (**B**) METTL3, (**C**) HNRNPC, (**D**) YTHDF1, (**E**) RBM15B, (**F**) HNRNPA2B1, (**G**) IGF2BP1, (**H**) ZC3H13, (**I**) ALKBH5, (**J**) YTHDC1, (**K**) VIRMA, (**L**) WTAP, (**M**) YTHDC2, (**N**) CBLL1, (**O**) METTL16, (**P**) IGF2BP2, (**Q**) YTHDF2, (**R**) RBM15, (**S**) YTHDF3, (**T**) METTL14, (**U**) FTO, (**V**) IGF2BP3 (*p < 0.05; **p < 0.01; ***p < 0.0001 compared with normal tissues).

By comparing the expression level of 22 m6A regulator genes in tumors with adjacent normal tissues, these seven genes were down-expression in tumor tissues: ZCCH4 (Fig. 4A), METTL3 (Fig. 4B), ZC3H13 (Fig. 4H), YTHDC1 (Fig. 4J), YTHDC2 (Fig. 4M), METTL16 (Fig. 4O), FTO (Fig. 4U), while those nine genes of HNRNPC (Fig. 4C), YTHDF1 (Fig. 4D), HNRNPA2B1 (Fig. 4F), IGF2BP1 (Fig. 4G), VIRMA(Fig. 4K), IGF2BP2 (Fig. 4P), YTHDF2 (Fig. 4Q), RBM15 (Fig. 4R), IGF2BP3 (Fig. 4V) were up-regulated in tumor tissues. Yellow background indicated the most significant (p < 0.0001) genes up-regulated in tumor tissues, including HNRNPC, YTHDF1, IGF2BP1, YTHDF2, RBM15, and IGF2BP3. Blue background indicated the most significant genes down-regulated in tumor tissues, including YTHDC1, YTHDC2, METTL16, and FTO. Through further analysis, the absolute expression of HNRNPC in tumor tissues was the highest, and the absolute expression of YTHDC2 was the lowest.

**Figure 5:**
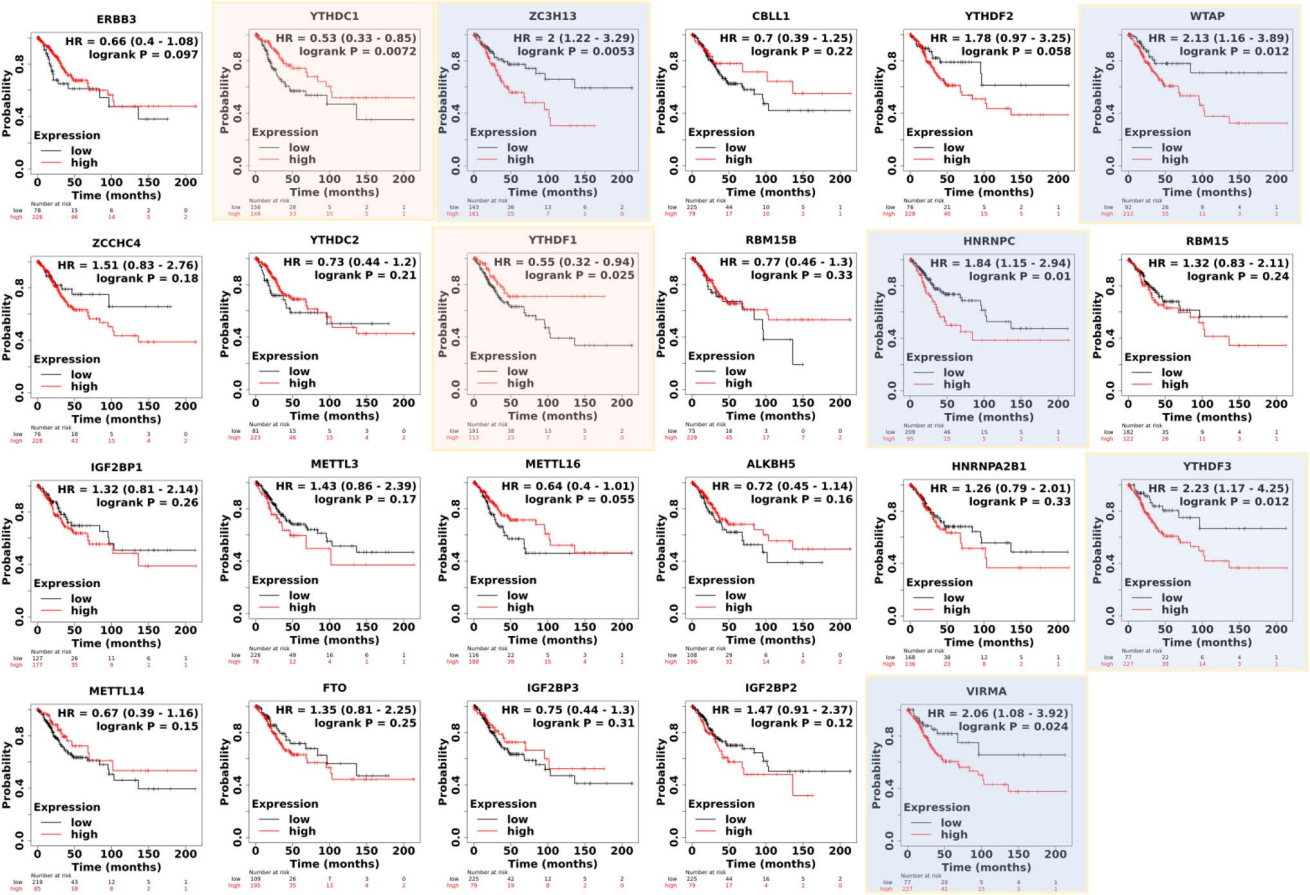
OS of the 22 m6A regulators and ERBB3 in cervical cancer.

In Fig. 5, we obtain the prognostic value of 22 m6A regulators in CESC through the Kaplan–Meier plot database. We revealed higher levels of ZC3H13, WTAP, HNRNPC, YTHDF3, and VIRMA were significantly associated with worse outcomes in CESC (HR > 1, blue background), while YTHDC1, YTHDF1 were protective factors of cervical cancer (HR < 1, orange background). It indicates these m6A regulators had key roles in CESC prognosis.

### Relationship between ERBB3 and m6A regulators

**Figure 6:**
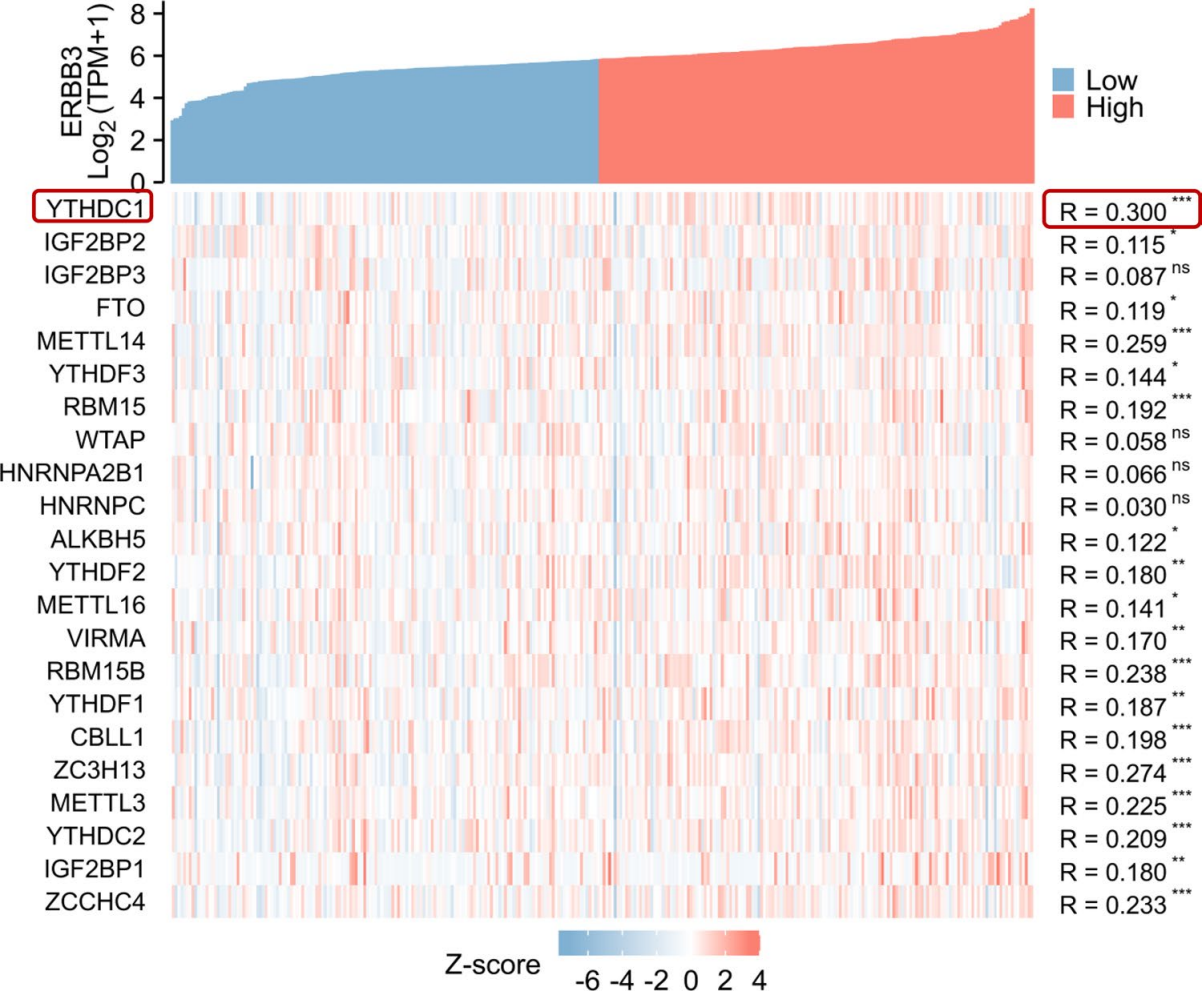
The correlation between the high and low expression of ERBB3 gene and m6A regulators in cervical cancer (ns: p ≥ 0.05; *p < 0.05; **p < 0.01; ***p < 0.001).

The upper part of Fig. 6 is the expression of ERBB3 gene, the lower part is the expression of m6A regulator genes after Z**-**score transformation. The thermal maps show ERBB3 expression has the most significant relation with YTHDC1, METTL14, RBM15, RBM15B, CBLL1, ZC3H13, METTL3, YTHDC2, and ZCCHC4. ERBB3 had the highest correlation with YTHDC1.

**Figure 7:**
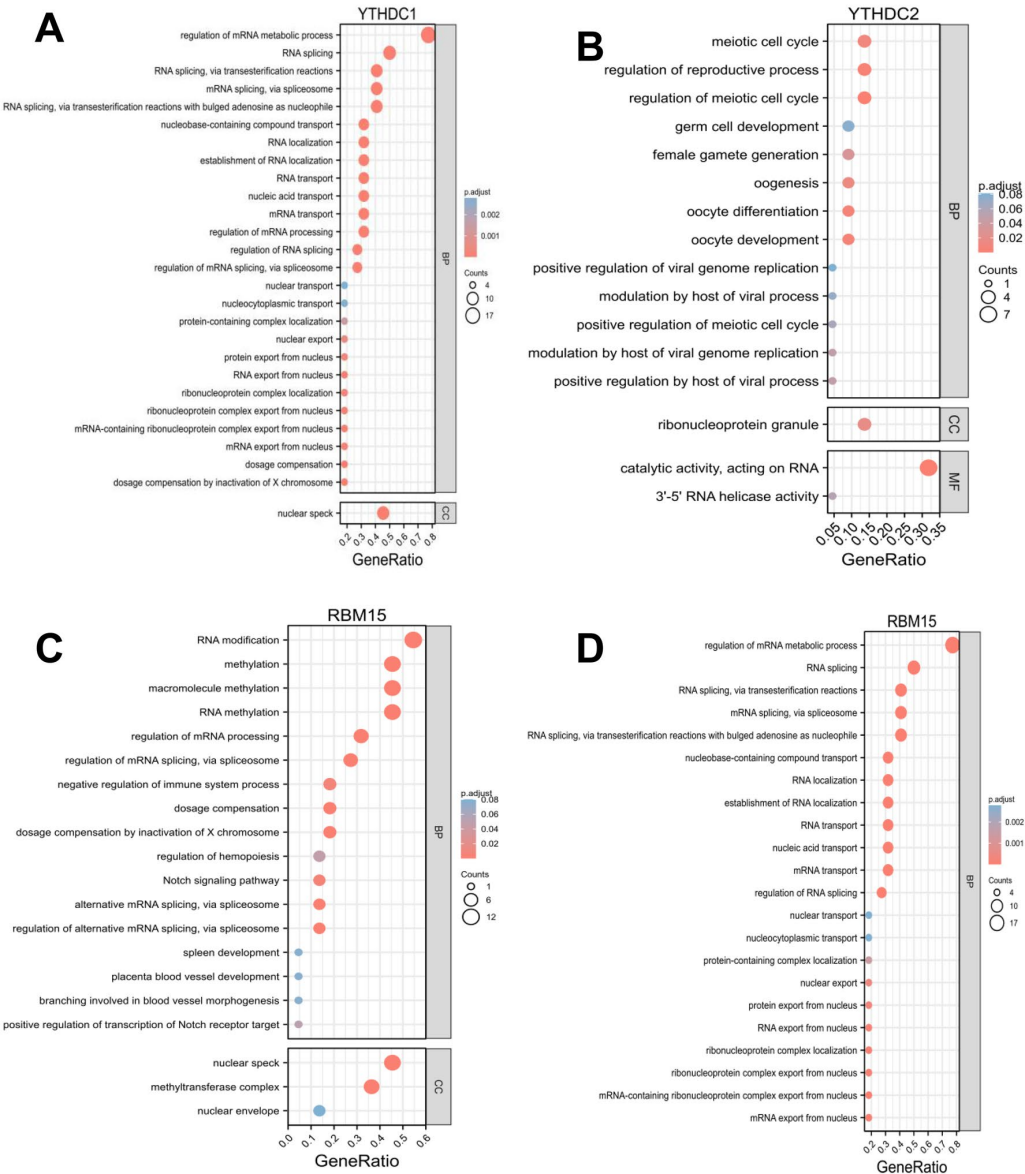
GO and KEGG enrichments of YTHDC1 (**A**), YTHDC2 (**B**), RBM15 (**C,D**) in CESC.

For the sake of investigating the downstream pathways of hub m6A regulators in CESC, we performed GO and KEGG analysis using co-expression genes of 6 m6A regulators. The results showed that YTHDC1 (Fig. 7A) and YTHDC2 (Fig. 7B) belong to RNA binding protein families. Both YTHDC1 and YTHDC2 can recognize and bind RNA containing N6 methyladenosine (m6A); The YTH domain mediates this binding^38–40^. M6A is a modifier existing in mRNA and some non-coding RNA internal sites, and plays a role in mRNA splicing, processing and stability regulation. YTHDC1 (also known as splicing factor yt521) can be used as a key regulator of exon addition or exon skipping to regulate selective splicing. YTHDC1 can promote exon increase by recruiting srsf3 into the region containing m6A, and inhibit exon skipping by blocking srsf10 binding to these same regions^20^. RBM15 (Fig. 7C,D) plays a major role in RNA modification and mRNA metabolic regulation.

**Figure 8:**
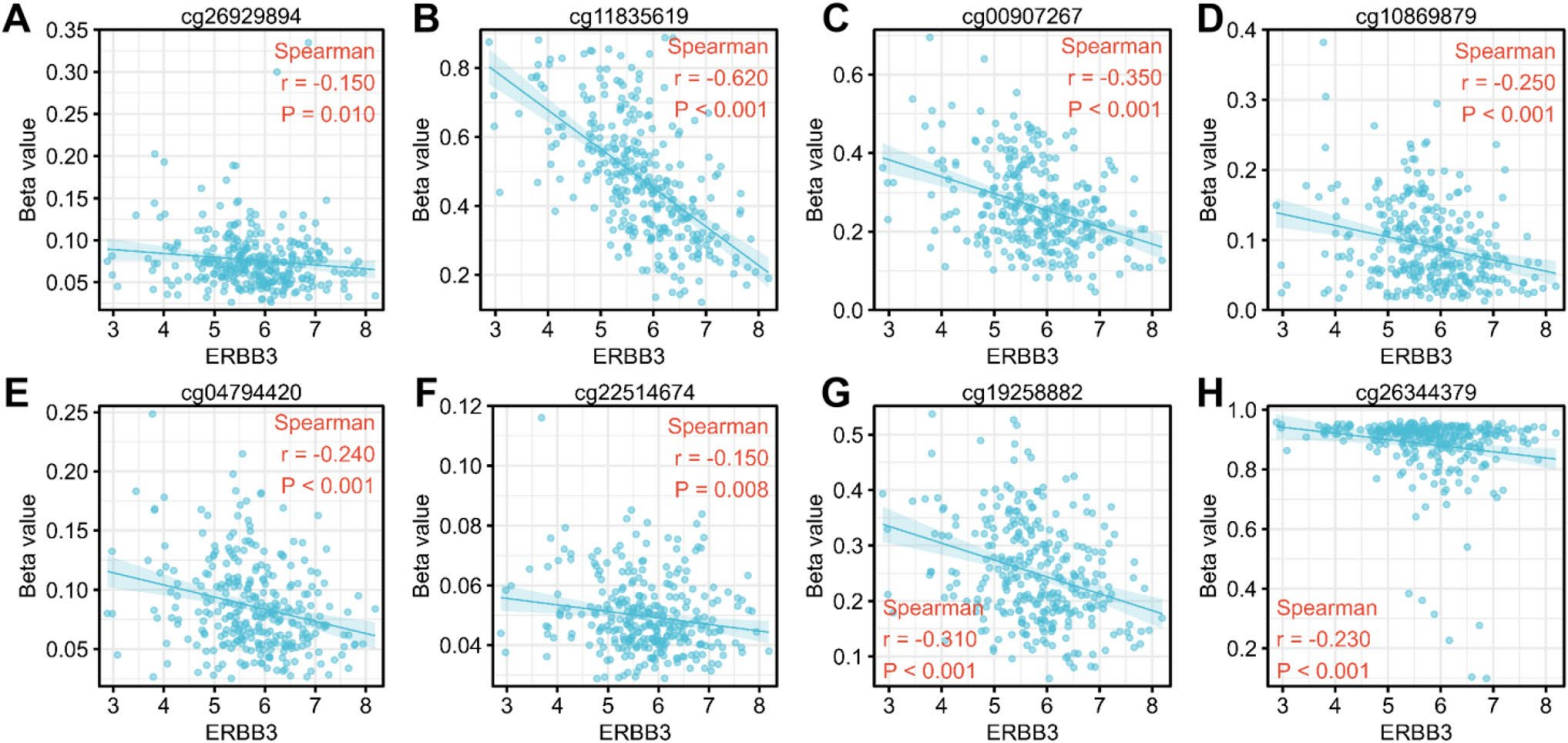
TPM values of ERBB3 in RNAseq data and Beta value corresponding to different methylation probe cg26929894 (**A**), cg11835619 (**B**), cg00907267 (**C**), cg10869879 (**D**), cg04794420 (**E**), cg22514674 (**F**), cg19258882 (**G**), cg26344379 (**H**). The beta value is the ratio of the methylated probe intensity to the overall intensity (the sum of the methylated and unmethylated probe intensities).

Figure 8 shows ERBB3 DNA methylation site using different methylation probe cg26929894 (TSS + 2290) (Fig. 8A), cg11835619 (TSS + 4480) (Fig. 8B), cg00907267 (TSS + 4187) (Fig. 8C), cg10869879 (TSS + 3985) (Fig. 8D), cg04794420 (TSS + 3808) (Fig. 8E), cg22514674 (TSS + 3159) (Fig. 8F), cg19258882 (TSS + 2221) (Fig. 8G), cg26344379 (TSS + 2033) (Fig. 8H). Beta value is a data format for estimating the degree of methylation. It represents the ratio of signal intensity between methylated and unmethylated bases. Beta values are usually between 0 and 1, 0 for no methylation and 1 for complete methylation. The probe of cg11835619 (TSS + 4480) has the highest relation with ERBB3 DNA methylation (r = 0.62, p < 0.001). Correlation analysis only shows that a methylation site regulates the correlation degree of ERBB3 gene, but further experiments (artificially regulating this methylation site to detect whether the expression of this gene has changed) must be done to clarify the causality.

### ERBB3 methylation andtumor immunity in the microenvironment (TIME) classification

There are interactive regulatory mechanisms in the local immune microenvironment of cervical cancer tumors. Cells, related cytokines, and immune effector cells have different distribution patterns and infiltration densities. We studied the relationship between ERBB3 DNA methylation modification and the infiltration of various immune components in the tumor immune microenvironment (TILS, Immunomodulator and Chemokine), and explored the possible mechanism of ERBB3 shaping the immune environment of cervical cancer based on DNA methylation.

#### TILs

Correlation between ERBB3 methylation and three different kinds of CD8 cells in CESC: Effector memory CD8 T cell (Tem_CD8) r = 0.625 (p < 0.01), Activated CD8 T cell(Act_CD8) r = 0.538 (p < 0.01), Central memory CD8 T cell (Tcm_CD8) r = 0.391 (p < 0.01) (Low correlation); Correlation between ERBB3 methylation and three different kinds of B cells in CESC: Activated B cell (Act_B) r = 0.658 (p < 0.01), Immature B cell (Imm_B) r = 0.684 (p < 0.01), Memory B cell (Mem_B) r = 0.121 (p < 0.01) (Low correlation); Correlation between ERBB3 methylation and Type 1T helper cell (Th1), Myeloid derived suppressor cell (MDSC), Macrophage, Regulatory T cell(Treg) in CESC, respectively. Th1 r = 0.683 (p < 0.01), MDSC r = 0.647 (p < 0.01), Macrophage r = 0.634 (p < 0.01), Treg r = 0.611 (p < 0.01). ERBB3 methylation is closely related to a variety of immune cells and factors in the tumor microenvironment, especially tumor-infiltrating lymphocytes. It can be seen from Table 2 that the TH1 and Immature B cell have a high correlation with ERBB3 methylation. It helps cells are mainly used to fight the immune response of intracellular bacteria and protozoa. They are mainly induced by interleukin 12 (IL-12). The main cytokine for execution is interferon gamma (IFN-γ).

**Table 2:** Spearman correlations between Methylation (met) of ERBB3 and TILs, immunomodulators, chemokines and receptors across CESC.

	Factor	Rho (P < 0.01)		Factor	Rho (P < 0.01)		Factor	Rho (P < 0.01)
Immuno-I	TIGIT	0.656	Immuno-S	ICOS	0.702	MHC	HLA-E	0.514
	CTLA4	0.642		CD48	0.659		HLA-QA2	0.471
	CD96	0.605		IL2RA	0.648		TAP1	0.471
	BTLA	0.599		CD27	0.626		HLA-DPB1	0.464
	HAVCR2	0.57		CD28	0.607		HLA-DRA	0.455
	CD244	0.558		CD86	0.604		HLA-PA1	0.454
	PDCD1	0.557		KLRK1	0.591			
				TNFRSF8	0.585			
				TNFSF13B	0.576			
				LTA	0.571			
TILS	Tem_CD8	0.625	CK	CXCL9	0.602	R	CXCR5	0.671
	Act_CD8	0.538		CXCL5	0.596		CCR5	0.656
	Tcm_CD8	0.391^a^		CXCL13	0.594		CCR7	0.62
	Act_B	0.658		CXCL11	0.56		CXCR6	0.618
	Imm_B	0.684		CCL19	0.476^a^		CXCR3	0.609
	Mem_B	0.121^a^		CCL18	0.467^a^		CCR2	0.589
	Th1	0.683		CCL13	0.45^a^			
	MDSC	0.647		*CCL21*	0.425^a^			
	Macrophage	0.634		*CCL22*	0.4^a^			
	Treg	0.611						

*Immuno-I* immunoinhibitor, *Immuno-S* immunostimulator, *MHC* MHC molecule, *TILs* tumor-infiltrating lymphocytes, *CK* chemokines, *R* receptors, *Tem_CD8* effector memory CD8 T cell, *Act_CD8* Activated CD8 T cell, *Tcm_CD8* central memory CD8 T cell, *Imm_B* immature B cell, *Mem_B* memory B cell, *MDSC* myeloid-derived suppressor cells, *Treg* regulatory T cells.

^a^Spearman correlations r < 0.5.

#### Immunoinhibitor, immunostimulator, MHC

The correlation coefficients between ERBB3 methylation and the abundance of immunoinhibitors in cervical cancer are arranged as follows: TIGIT r = 0.656 (p < 0.01), CTLA4 r = 0.642 (p < 0.01), CD96 r = 0.605 (p < 0.01), BTLA r = 0.599 (p < 0.01), HAVCR2 r = 0.57 (p < 0.01), CD244 r = 0.558 (p < 0.01), PDCD1 r = 0.557 (p < 0.01), PDCD1LG2 r = 0.548 (p < 0.01), LAG3 r = 0.519 (< 0.01). The correlation coefficients between ERBB3 methylation and the abundance of immunostimulators in cervical cancer are arranged as follows: ICOS r = 0.702 (p < 0.01), CD48 r = 0.659 (p < 0.01), IL2RA r = 0.648 (p < 0.01), CD27 r = 0.626 (p < 0.01), CD28 r = 0.607 (p < 0.01), CD86 r = 0.604 (p < 0.01), KLRK1 r = 0.591 (p < 0.01), TNFRSF8 r = 0.585 (p < 0.01), TNFSF13B r = 0.576 (p < 0.01), LTA r = 0.571 (p < 0.01). The correlation coefficients between ERBB3 methylation and the abundance of MHC in cervical cancer are arranged as follows: HLA-E r = 0.514 (p < 0.01), HLA-DQA2 r = 0.471 (p < 0.01), TAP1 r = 0.471 (p < 0.01), HLA-DPB1 r = 0.464 (p < 0.01), HLA-DRA r = 0.455 (p < 0.01), HLA-DPA1 r = 0.454 (p < 0.01). TIGIT inhibits T cell activation by promoting the production of mature immunomodulatory dendritic cells. ICOS enhances all basic responses of T cells to foreign antigens, namely proliferation, secretion of lymphokines, up regulation of molecules mediating cell–cell interactions, and effectively help B cells secrete antibodies.

#### Chemokine and receptors

In CESC, the order of chemokines associated with ERBB3 methylation from high to low is as follows: CXCL9 (r = 0.602, p < 0.01), CXCL5 (r = 0.596, p < 0.01), CXCL13 (r = 0.594, p < 0.01), CXCL11 (r = 0.56, p < 0.01), CCL19 (r = 0.476, p < 0.01), CCL18 (r = 0.467, p < 0.01), CCL13 (r = 0.45, p < 0.01), CCL21 (r = 0.425, p < 0.01), CCL22 (r = 0.4, p < 0.01); The order of Receptor associated with ERBB3 methylation from high to low is as follows: CXCR5 (r = 0.671, p < 0.01), CCR5 (r = 0.656, p < 0.01), CCR7 (r = 0.62, p < 0.01), CXCR6 (r = 0.618, p < 0.01), CXCR3 (r = 0.609, p < 0.01), CCR2 (r = 0.589, p < 0.01). (External Links include HGNC, NCBI, Ensembl, Uniprot, GeneCards data base). CXCL9 affects the growth, movement, or activation state of cells that participate in immune and inflammatory response. CXCR5 is expressed in mature B-cells, involved in B-cell migration.

## Discussion

ERBB receptor is a typical cell membrane receptor tyrosine kinase, which is activated by dimerization after binding to ligands. The ERBB receptor tyrosine kinase family contains four cell surface receptors: ERBB1/EGFR/HER1, ERBB2/HER2, ERBB3/HER3 and ERBB4/HER4. HER3/ERBB3 is a member of the ERBB receptor protein tyrosine kinase family, but lacks tyrosine kinase activity. The tyrosine phosphorylation of ERBB3 depends on its binding to other ERBB tyrosine kinases. When binding to ligands, heterodimers were formed between ERBB3 and other ERBB proteins, and ERBB3 was phosphorylated by activated ERBB kinase on tyrosine residues^41,42^. There are at least nine potential tyrosine phosphorylation sites in the tail region of carboxyl end of ERBB3. These sites are the common binding sites of signal transduction proteins (including Src family members, Grb2 and PI3 kinase p85 subunit), which can mediate downstream signal transduction of ERBB^43^. The Tyr1222 and Tyr1289 sites of ERBB3 are located in the YXXM motif and participate in PI3K signal transduction^44^. Researchers have found that ERBB3 is highly expressed in many cancer cells^45^.

In this study, it was found that in ERBB family, the expression of ERBB3 in cervical cancer tissues was higher than that in normal tissues (Fig. 1A). It shows that ERBB3 may bes closely related to adenocarcinoma and HPV positive cervical carcinoma (Figs. 1B, 2B).

Figure 1F shows the comparison of transcriptome expression of four ERBB families in cervical cancer tissues in Table 1. It is found that the high expression of ERBB3 in tumor tissues is consistent with the high expression of ERBB2. However, ERBB3 had no statistical significance in clinical disease staging and disease prognosis (Figs. 1D,E,G, 2E). Therefore, we speculate that ERBB3 is a pathogenic factor of cervical cancer rather than a prognostic factor. The hotspot mutation site of ERBB3 in cervical cancer is extracellular domain V104M/L (Fig. 2A,C), the one has been shown to be a statistically significant mutation hotspot to promote oncogenic signalling^46^. Treatments of cells harboring V104M mutation in ERBB3 with ERBB antibodies and other inhibitors blocked oncogenic signaling^47^.

Abnormal methylation is a prominent feature of cancer. It is unclear how DNA methylation affects immune surveillance and tumor metastasis. N6-methyladenosine (m6A) is one of the most common and representative chemical modifications in eukaryotic RNA. It is a kind of dynamic and heritable information, which is widely present in a variety of organisms. M6A is mainly divided into its important roles in regulating gene expression, splicing, RNA editing, regulating RNA stability, and controlling mRNA degradation. It is a reversible epigenetic modification. Mainly divided into three categories: m6A methyltransferase (writer), m6A demethylase (eraser), m6A binding protein (reader).

Among them, we found higher expressions of HNRNPC, YTHDF1, IGF2BP1, YTHDF2, RBM15, and IGF2BP3, lower expression of YTHDC1, YTHDC2, METTL16, and FTO in CESC (Fig. 4). YTHDC1 and YTHDF1 have anti-cancer effects in cervical cancer, while ZC3H13, WTAP, HNRNPC, YTHDF3 and VIRMA have tumor-promoting effects in cervical cancer (Fig. 5). We compared the methylation regulatory factors with the highest correlation in Figs. 5 and 6, and obtained three factors in Fig. 7. Through further GO and KEGG clustering analysis of the functions of these three factors (YTHDC1, YTHDC2 and RBM15), we infer that ERBB3 methylation plays an important role in the occurrence and progression of cervical cancer.

According to the analysis of ERBB3 DNA methylation in Fig. 8, in cervical cancer, the correlation of DNA methylation modification of the 4480 base pair downstream of ERBB3 transcription initiation site was the highest, but whether the gene was finally regulated still needs further experiments to determine the causal relationship.

GO and KEGG analysis confirm ERBB3 Methylation were involved in regulating RNA splicing, RNA stability, and cell proliferation create Tumor and immune system interaction, which integrates multiple heterogeneous data types (Fig. 7).

We researched the relationship between ERBB3 methylation and immune cell infiltration in cervical cancer microenvironment, finding that (Table 2) the abundance of TH1, MDSC, Macrophage, effector memory CD8 T cell, activated CD8 T cell, immature B cell and regulatory T cell have the significant association with methylation of ERBB3 in cervical tumor immune microenvironment (R > 0.6). Relations between three kinds of immunomodulators and methylation of ERBB3 are as follows: TIGIT (r = 0.656), ICOS (r = 0.702), and HLA-E (r = 0.514). TIGIT (T cell Ig and ITIM domain) is a member of the poliovirus receptor (PVR)/Nectin family^48^. It is expressed in lymphocytes, especially effector and regulatory CD4^+^ T cells, follicular auxiliary CD4^+^ T cells, effector CD8^+^ T cells and natural killer (NK) cells. TIGIT plays an inhibitory role in multiple steps of the tumor immune cycle^49^. The immune regulation of ICOS is manifested in the following aspects: enhancing the pattern recognition receptor signal of dendritic cells, inducing CD4^+^ T cells to produce IL-10 and producing high affinity antibodies against specific antigens.

CXCR5 (r = 0.671) is a G protein-coupled seven transmembrane receptor, belonging to the CXC chemokine receptor family^50^, and its ligand is the chemokine CXCL13 (r = 0.594). CCR5 (r = 0.656) is the receptor of intracellular β-chemokines (RANTES, MIP1α and MIP1β), which has the function of regulating the migration, proliferation and immunity of T cells and monocytes/macrophage cell lines^51^. It is mainly expressed in the memory quiescent T Lymphocytes, monocytes, immature dendritic cells, etc. on the cell membrane. When cancer cells spread in the body, secondary tumors called metastases can form. These secondary tumors cause approximately 90% of cancer patients’ deaths. An important way to spread cancer cells is through the lymphatic system, which runs through the entire body like the vascular system and connects the lymph nodes to each other. When white blood cells migrate through the lymphatic system to coordinate defenses against pathogens, as a specific membrane protein, chemokine receptor 7 (CCR7)^52^ (r = 0.62) plays an important role. It is located in the cell membrane, and it can receive external signals and transmit these signals to the inside of the cell. The receptor for CXCL9 (r = 0.602) (also known as IFN-r) is CXCR3 (r = 0.609) of the CXC subfamily^53^.

These results demonstrate that ERBB3 dynamically reshapes the composition and function of the immune macroenvironment in cervical cancer. The correlations between methylation and TIME shows potential correlation between methylation of ERBB3 and inhibitory immune checkpoints along with immunosuppressive Tregs, MDSCS and potentially macrophages indicates an immune permissive environment favoring tumors to grow. Chemokines CXCL19 and chemokine receptors CXCR5 mediate immune cell trafficking into the tumour micro environment based on the DNA methylation of ERBB3 in cervical cancer.

## Conclusions

In summary, this work proves that ERBB3 gene mutation, methylation modification have extensive regulatory mechanisms on the CESC microenvironment. ERBB3 is more likely to be an important carcinogenic factor of cervical cancer, but it has no significant effect on the clinical stage and prognosis of the disease. The differences in methylation modification patterns lead to the heterogeneity and complexity of CESC tumor immune microenvironment. Our study found that m6A regulator was an important biomarker of CESC and was closely related to tumor immune infiltration in cervical cancer. A comprehensive assessment of the ERBB3 multi-omics will help to enhance our understanding of the characteristics of cell infiltration in CESC tumor microenvironment and guide more effective immunotherapy strategies. DNA methylation modification of the 4480 base pair downstream of ERBB3 transcription initiation site was the highest. Further experiments will verify the carcinogenic mechanism of this methylation site on ERBB3 in cervical cancer.

## Data Availability

The raw data required to reproduce these findings cannot be shared at this time as the data also forms part of an ongoing study.
